# Polysaccharides from *Trifolium repens* L. extracted by different methods and extraction condition optimization

**DOI:** 10.1038/s41598-019-42877-5

**Published:** 2019-04-23

**Authors:** Hongmei Shang, Ran Li, Hongxin Wu, Zewei Sun

**Affiliations:** 1 College of Animal Science and Technology, Jilin Agricultural University, Changchun, 130118 China; 2 Key Laboratory of Animal Nutrition and Feed Science of Jilin Province, Changchun, 130118 China; 3 Key Laboratory of Animal Production, Product Quality and Security, Ministry of Education, Changchun, 130118 China; 4 Grassland Research Institute of CAAS, Hohhot, 010010 China

**Keywords:** Polysaccharides, Isolation, separation and purification

## Abstract

Four different extraction methods, including hot water extraction (HWE), ultrasonic-assisted extraction (UAE), enzyme-assisted extraction (EAE) and ultrasonic-enzyme-assisted extraction (UEAE), were applied to extract polysaccharides from *Trifolium repens* L. (TRPs). In addition, response surface methodology (RSM) was performed to optimize the extraction conditions of TRPs. The results showed that different extraction methods had significant effects on the extraction yields and antioxidant activities of TRPs. TRPs extracted by the EAE method (10.57%) and UEAE method (10.62%) had significantly higher extraction yields than TRPs extracted by the HWE method (8.35%) and UAE method (9.43%) (*P* < 0.05). However, there was no significant difference between the extraction yields of the EAE method and UEAE method (*P* > 0.05). TR*P*s extracted by the EAE method had a higher content of uronic acid and exhibited better antioxidant capacities. Therefore, EAE was selected as the optimal extraction method to extract TRPs. The optimal extraction conditions of EAE to extract TPRs were liquid–solid ratio 30 mL/g, enzymolysis time 87 min, enzyme-complex dosage 1.6% and pH 6, leading to a TRPs yield of 13.15%.

## Introduction

The *Trifolium genus* contains approximately 240 species of clovers^1^, mainly distributed in western North America, the Mediterranean basin and the highlands of eastern Africa^2^. Some *Trifolium* species, such as *Trifolium repens* L. and *Trifolium pratense* L., have been used as medicinal plants, forages and landscaping plants in many countries^3^. *Trifolium repens* L. has a large biomass and strong adaptability, producing 45000–60000 kg of fresh grass per hectare with mowing tolerance and good regeneration^4^. Polysaccharides are a type of polymer and exhibit diverse bioactivities, such as antioxidant^5^, antitumor^6^, anti-inflammation^7^, and immunoregulation^8^ properties. Using polysaccharides resources may help to broaden the utilization of *Trifolium repens* L.

In a previous study, water extraction was used to extract polysaccharides from *Trifolium repens* L., and the effects of three drying methods (hot air drying, freeze drying and vacuum drying) on the physical and chemical properties and antioxidant activities of polysaccharides were compared, so as to screen out the best drying methods for polysaccharides from *Trifolium repens* L^9^. Ouyang K. H. *et al*. conducted an experiment to study the optimal water extraction condition for polysaccharides from white clover using a single-factor experiment and an L_9_ (3^4^) orthogonal experiment^10^. Actually, extraction techniques had significant effects on the extraction yields, physicochemical characteristics and bioactivities of polysaccharides^11^. Recently, various extraction techniques, such as hot water extraction (HWE)^12^, microwave-assisted extraction^13^, ultrasonic-assisted extraction (UAE)^14^, enzyme-assisted extraction (EAE)^15^, ultrasonic-enzyme-assisted extraction (UEAE)^16^, and smashing tissue extraction^17^^,^ have been used to extract polysaccharides. Nevertheless, to the best of our knowledge, research on the physicochemical characteristics and the bioactivities of polysaccharides from *Trifolium repens* L. (TRPs) using different techniques has not been conducted to date.

Response surface methodology (RSM) can be used to obtain the optimal extraction conditions of polysaccharides, while Box-Behnken design (BBD) is more efficient compared to other methods due to fewer runs^18^. In this study, TRPs were extracted by four methods, that is, HWE, UAE, EAE, and UEAE. The physicochemical properties and antioxidant activities of TRPs were determined to select the suitable extraction method. In addition, the processing parameters for the suitable extraction method selected were optimized by RSM. The final purpose of this study is to provide a basis for the development and utilization of *Trifolium repens* L.

## Results and discussion

### Extraction yield, pH, solubility and chemical composition of TRPs extracted by different methods

As shown in Table 1, the yields of the four TRPs were measured as HWE-TRPs (8.35%) < UAE-TRPs (9.43%) < EAE-TRPs (10.57%) < UEAE-TRPs (10.62%). Uronic acid is one of the active parts of polysaccharides, and the higher content of uronic acid might suggest higher bioactivities of polysaccharides^19^. The uronic acid contents were significantly different in the following order: HWE-TRPs (4.04%) < UAE-TRPs (4.27%) < UEAE-TRPs (5.17%) < EAE-TRPs (5.42%) (*P* < 0.05). There were no significant differences on the extraction yields and uronic acid contents between the EAE method and UEAE method (*P* > 0.05). However, TRPs extracted by the EAE method and UEAE method had significantly higher extraction yields and uronic acid contents than TRPs extracted by the HWE method and UAE method (*P* < 0.05). In the process of polysaccharide extraction, improving the penetration of solvent into cells is the key factor to facilitate the extraction process. HWE could accelerate improve the extraction efficiency with the increased temperature of water. The extraction process can also be further facilitated by physical methods, such as UAE, EAE and UEAE, they can largely promote the dissolution of polysaccharides through biodegradation or mechanical destruction of plant cell walls^20^. The results in the present study indicated that the EAE and UEAE methods could significantly improve the TRPs yields and uronic acid contents compared to the HWE method and UAE method (*P* < 0.05), possibly because the enzyme complex (cellulase, papain and pectinase) can, separately or in conjunction with ultrasound, facilitate TRPs into the extraction solvents through enzymatic hydrolysis and cavitation effects. A similar result was also reported by Li and Wang^21^.

**Table 1:** Chemical composition of TRPs extracted by different extraction methods.

Samples	HWE-TRPs	UAE-TRPs	EAE-TRPs	UEAE-TRPs
Polysaccharides yield (%)	8.35 ± 0.36^c^	9.43 ± 0.14^b^	10.57 ± 0.36^a^	10.62 ± 0.22^a^
Total polysaccharides content (%)	83.60 ± 0.41^a^	84.42 ± 0.93^a^	84.73 ± 1.27^a^	83.76 ± 1.28^a^
Uronic acid content (%)	4.04 ± 0.18 ^b^	4.27 ± 0.50^b^	5.42 ± 0.07^a^	5.17 ± 0.13^a^
Moisture content (%)	8.45 ± 0.15^a^	8.49 ± 0.14^a^	8.54 ± 0.27^a^	8.19 ± 0.12^a^
pH	7.04 ± 0.01^a^	7.03 ± 0.01^a^	7.03 ± 0.01^a^	7.05 ± 0.02^a^

Values are presented as mean ± standard deviation (n = 3). Different letters within the same row show significant difference (*P* < 0.05).

There were no significant differences in the contents of total polysaccharides and moisture or the pH of the four TRPs (*P* > 0.05). Protein was not detected in the four TRPs. No significant differences were found in the solubility time of the four TRPs at each determination temperature (*P* > 0.05) (Table 2). The solubility time of HWE-TRPs, UAE-TRPs, EAE-TRPs and UEAE-TRPs at 20 °C was 16.47 s, 15.77 s, 15.87 s and 16.13 s, respectively.

**Table 2:** Solubility time of TRPs extracted by different extraction methods.

Samples	HWE-TRPs	UAE-TRPs	EAE-TRPs	UEAE-TRPs
20 °C (s)	16.47 ± 0.31^a^	15.77 ± 0.96^a^	15.87 ± 0.59^a^	16.13 ± 0.47^a^
40 °C (s)	13.20 ± 0.53^a^	12.73 ± 0.21^a^	12.70 ± 0.17^a^	12.60 ± 0.46^a^
60 °C (s)	11.10 ± 0.56^a^	10.73 ± 0.21^a^	11.03 ± 0.25^a^	10.83 ± 0.35^a^
80 °C (s)	7.77 ± 0.15^a^	7.57 ± 0.21^a^	7.73 ± 0.32^a^	7.50 ± 0.40^a^
100 °C (s)	5.27 ± 0.50^a^	5.40 ± 0.35^a^	5.07 ± 0.25^a^	5.27 ± 0.21^a^

Values are presented as mean ± standard deviation (n = 3). Different letters within the same row show significant difference (*P* < 0.05).

### Molecular weight distribution of TRPs extracted by different methods

**Figure 1:**
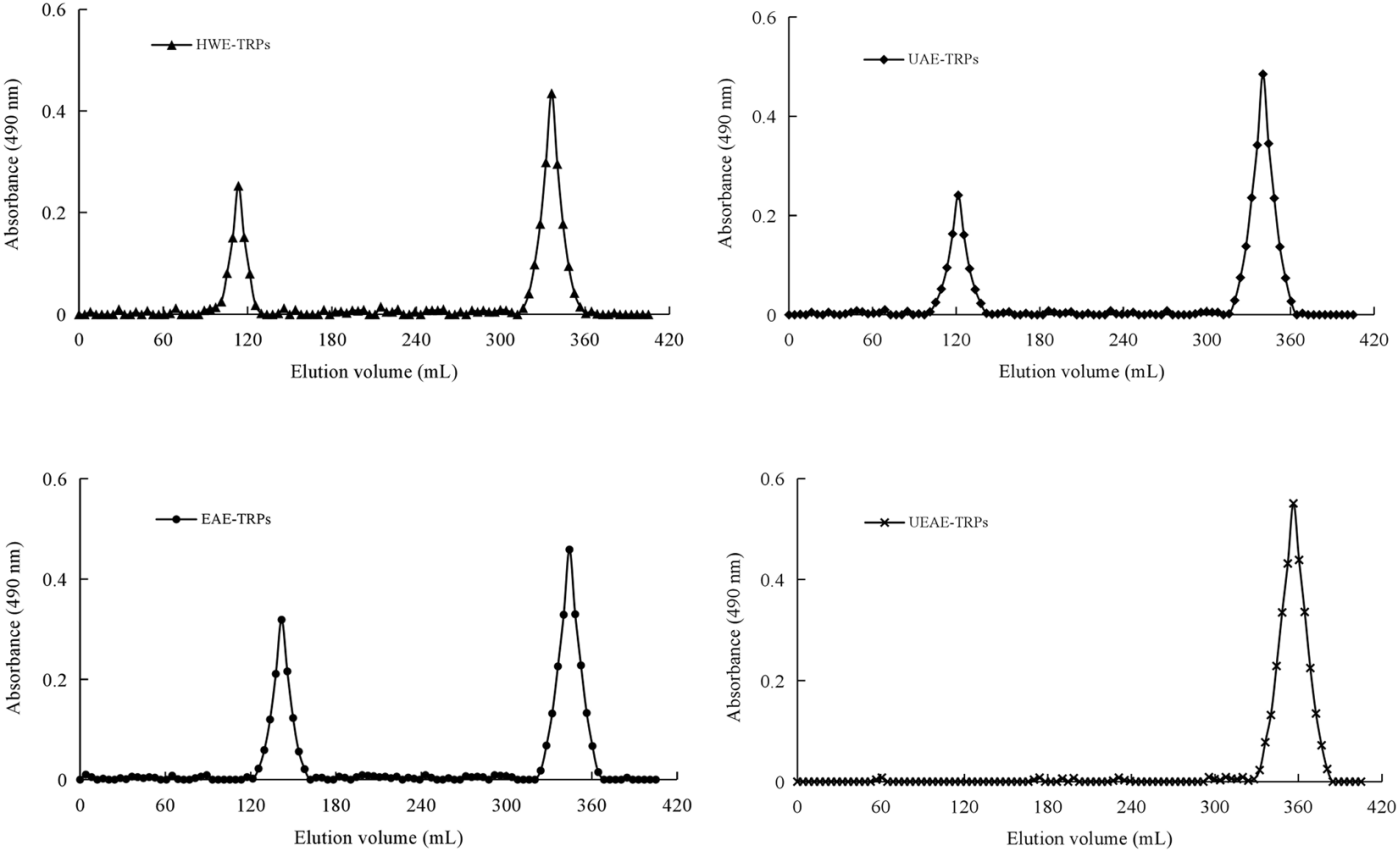
Molecular weight distribution of TRPs extracted by different extraction methods.

Molecular weight is an important property of polysaccharides. Different molecular weights affect the physicochemical properties and bioactivities of polysaccharides^19^. The molecular weight distributions of the four TRPs were presented in Fig. 1. UEAE-TRPs showed one distinct group with a molecular weight of 2500.99 Da. However, there were two distinct groups of molecular weight distribution for the other three TRPs: HWE-TRPs showed molecular weights of 1147.66 × 10^4^ Da and 5049.56 Da; UAE-TRPs showed molecular weights of 866.47 × 10^4^ Da and 4387.58 Da; EAE-TRPs showed molecular weights of 429.15 × 10^4^ Da and 3812.39 Da. These results suggested that the UEAE, EAE and UAE techniques could obtain TRPs with lower molecular weights than the HWE method. The higher molecular weight of HWE-TRPs indicated that high temperature easily caused the aggregation of polysaccharides. Long time ultrasonic extraction would destroy the molecular chains of polysaccharides and degrade polysaccharide molecules^22^. In addition, enzyme can degrade the polysaccharides to some degree^23^. The lower molecular weight of UEAE-TRPs among the four TRPs might be due to the synergistic effect of ultrasonic and enzyme complexes (cellulase, papain and pectinase), which could decompose the TRPs to form smaller ones, and finally, UEAE-TRPs showed one distinct group. This result was consistent with the report of Li and Wang^21^, who studied the impact of four extraction methods (hot water, enzyme assistance, ultrasonic assistance and ultrasonic-enzyme assistance) on the molecular weight of the *Hohenbuehelia serotina* polysaccharides (HSP) and found that UEA-HSP exhibited the largest distribution of molecular weight, which also might be observed because cellulase or ultrasound could decompose the polysaccharides to form small ones.

### Monosaccharide composition of TRPs extracted by different methods

**Figure 2:**
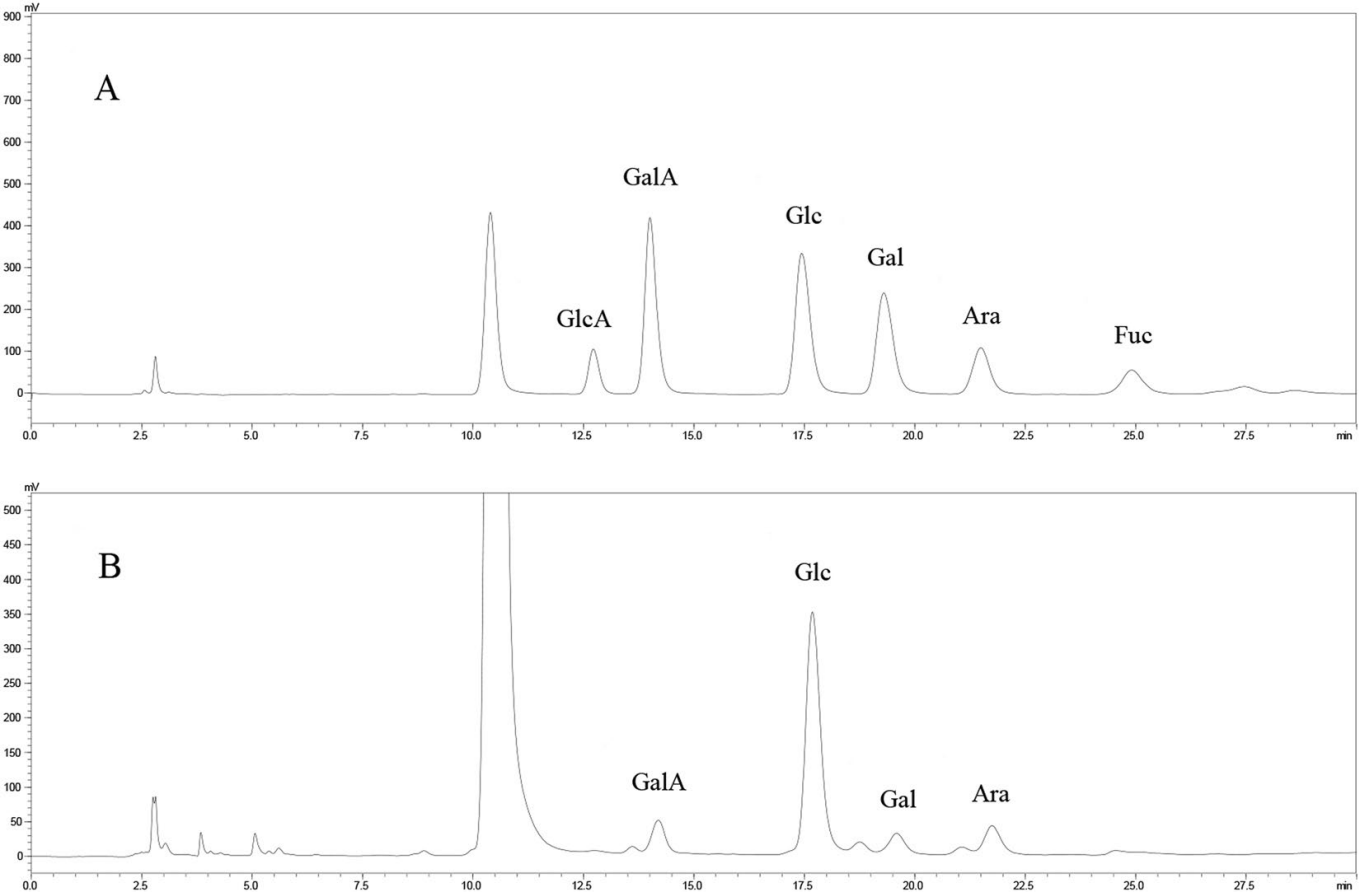
HPLC chromatogram for monosaccharide composition: (**A**) standard substances; (**B**) *Trifolium repens* L. polysaccharides. GlcA, Glucuronic acid; GalA, galacturonic acid; Glc, glucose; Gal, galactose; Ara, arabinose; Fuc, fucose.

HPLC analysis showed that the four TRPs were composed of galacturonic acid (GalA), glucose (Glc), galactose (Gal) and arabinose (Ara) (Fig. 2). The ratios of GalA, Glc, Gal and Ara in the four TRPs are shown in Table 3. The GalA content of EAE-TRPs (4.82%) was higher than that of HWE-TRPs (2.97%), UAE-TRPs (3.81%) and UEAE-TRPs (4.46%) (*P* < 0.05). The variations in the monosaccharide composition and proportion of polysaccharides were related with the differences in the extraction techniques and temperature^24,25^. In addition, monosaccharide composition is a primary factor in understanding the bioactivities of polysaccharides. The higher uronic acid content of polysaccharides might indicate its stronger biological activities^19^.

**Table 3:** Monosaccharide composition of TRPs extracted by different extraction methods.

Samples	HWE-TRPs	UAE-TRPs	EAE-TRPs	UEAE-TRPs
GalA (%)	2.97 ± 0.15^d^	3.81 ± 0.17^c^	4.82 ± 0.12^a^	4.46 ± 0.17^b^
Glc (%)	51.25 ± 1.01^a^	51.24 ± 0.81^a^	52.25 ± 0.82^a^	52.56 ± 1.35^a^
Gal (%)	23.11 ± 1.94^a^	22.10 ± 1.09^a^	21.98 ± 0.63^a^	23.25 ± 1.01^a^
Ara (%)	22.67 ± 2.79^a^	22.85 ± 0.26^a^	20.94 ± 1.21^a^	19.74 ± 2.25^a^

Values are presented as mean ± standard deviation (n = 3). Different letters within the same row show significant difference (*P* < 0.05). GalA, galacturonic acid; Glc, glucose; Gal, galactose; Ara, arabinose.

### Antioxidant activities of TRPs extracted by different methods

**Figure 3:**
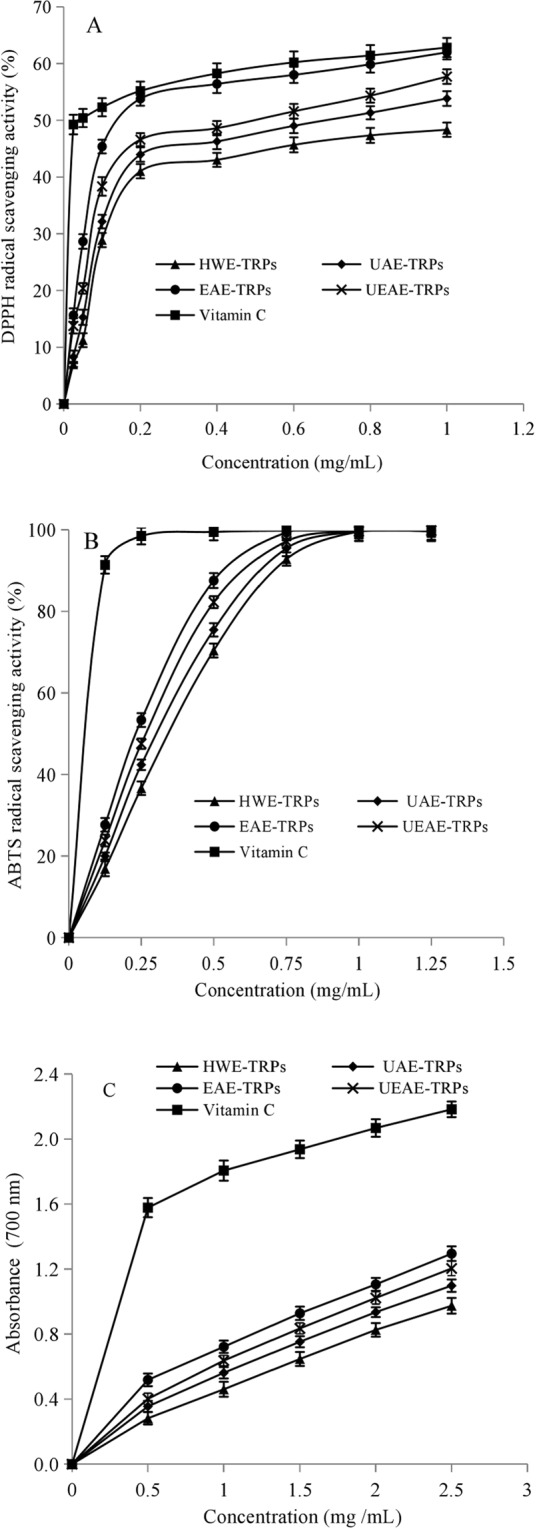
Antioxidant activities of TRPs extracted by different extraction methods: (**A**) DPPH radical scavenging activity; (**B**) ABTS radical scavenging activity; (**C**) Ferric reducing power. Error bars presented the standard deviationas of means (n = 3). Duncan’s post hoc test was used in inmultiple means comparison procedure.

The 1,1-diphenyl-2-picrylhydrazyl (DPPH) free radical is a stable free radical and can accept an electron or hydrogen radical to become a stable diamagnetic molecule, which has been widely accepted as a tool for estimating the free-radical scavenging activities of antioxidants^26^. The scavenging activities of the four TRPs on the DPPH radical were presented in Fig. 3A. The EAE-TRPs showed stronger DPPH scavenging ability compared with HWE-TRPs, UAE-TRPs and UEAE-TRPs (*P* < 0.05) at each polysaccharides concentration. The higher content of uronic acid in EAE-TRPs might contribute to its higher DPPH radical scavenging activity. Zhang *et al*. indicated that polysaccharides with higher uronic acid content had higher bioactivities^27^. The highest DPPH radical scavenging activity of EAE-TRPs (61.99%) was detected at 1 mg/mL, which was not significantly different from that of vitamin C (62.83%) (*P* > 0.05), indicating that EAE-TRPs had strong scavenging ability on DPPH radicals.

The 2,2-azino-bis-(3-ethyl-benzothiazoline-6-sulfonic acid) (ABTS) radical is a free and stable radical cation that can react with antioxidants through acceptance of a hydrogen atom or an electron^28^. The scavenging activities of the four TRPs on the ABTS radical were presented in Fig. 3B. The results showed that the ABTS radical scavenging activities of the four TRPs were in the following order: HWE-TRPs < UAE-TRPs < UEAE-TRPs < EAE-TRPs. Uronic acid could trigger the hydrogen atom of the anomeric carbon^29^. Therefore, the higher content of uronic acid in EAE-TRPs might contribute to its higher scavenging activity among the four TRPs. At the concentration of 1 mg/mL, the scavenging activities of the four TRPs were all more than 99.00%, reaching the scavenging ability of vitamin C. These results indicated that TRPs had strong ABTS scavenging ability.

The reducing power could directly reflect the donation of an electron or hydrogen and has been widely employed to investigate the antioxidant activities of natural compounds^28^. The ferric reducing powers of the four TRPs and vitamin C were presented in Fig. 3C. The ferric reducing power of TRPs was lower than that of vitamin C (*P* < 0.05), which was consistent with the findings of polysaccharides extracted from *Inonotus obliquus*^19^ and *Hohenbuehelia serotina*^28^ on reducing powers. Among the four TRPs, EAE-TRPs and UEAE-TRPs exhibited higher ferric reducing power than HWE-TRPs and UAE-TRPs, which might be due to their lower molecular weights. There are more exposed reducing ends in the polysaccharides with lower molecular weights than those with higher molecular weights. Therefore, the polysaccharides with lower molecular weights have higher ferric reducing powers^30^.

The antioxidant ability of polysaccharides might be related to their uronic acid content, monosaccharide composition and molecular weight^31^. Therefore, the antioxidant activities of polysaccharides are not a function of a single factor but a combination of several factors^32^. In the present study, TRPs obtained by EAE exhibited higher radical scavenging activities and reducing powers and were stronger than those of the other three types of TRPs, indicating that more bioactive polysaccharides could be extracted using the EAE method. The higher antioxidant activities of EAE-TRPs might due to its highest GalA content and lower molecular weight. Another possible mechanism may involve the degradation of polysaccharides and further changes in the chemical structures induced by enzymolysis treatment, which warrant further study.

### Extraction condition optimization of the EAE method to extract TRPs

According to the evaluation of the physicochemical characteristics and activities of TRPs extracted by different methods, EAE-TRPs presented higher extraction yields and antioxidant activities among the four TRPs. Therefore, EAE was selected as the optimum extraction method to extract TRPs. RSM was performed to obtain the optimal extraction parameters of EAE to extract TRPs.

### Single-factor-test analysis

To evaluate the effect of liquid-solid ratio on the TRPs yield, the extraction experiments were performed at different liquid-solid ratios (15, 20, 25, 30 and 35 mL/g) while enzymolysis time, enzyme-complex dosage and pH were set at 90 min, 1% and 6, respectively. To determine the effect of enzymolysis time (30, 60, 90, 120 and 150 min) on the TRPs yield, the other three factors, including liquid-solid ratio, enzyme-complex dosage and pH, were set to 25 mL/g, 1% and 6, respectively. To estimate the effect of enzyme-complex dosage (0.5, 1.0, 1.5, 2.0 and 2.5%) on the TRPs yield, the other three factors, including liquid-solid ratio, enzymolysis time and pH, were fixed at 25 mL/g, 90 min and 6, respectively. To measure the effect of pH on the TRPs yield, the extraction experiments were performed at different pH conditions (3, 4, 5, 6 and 7) while liquid-solid ratio, enzymolysis time and enzyme-complex dosage were set to 25 mL/g, 90 min, and 1%, respectively.

**Figure 4:**
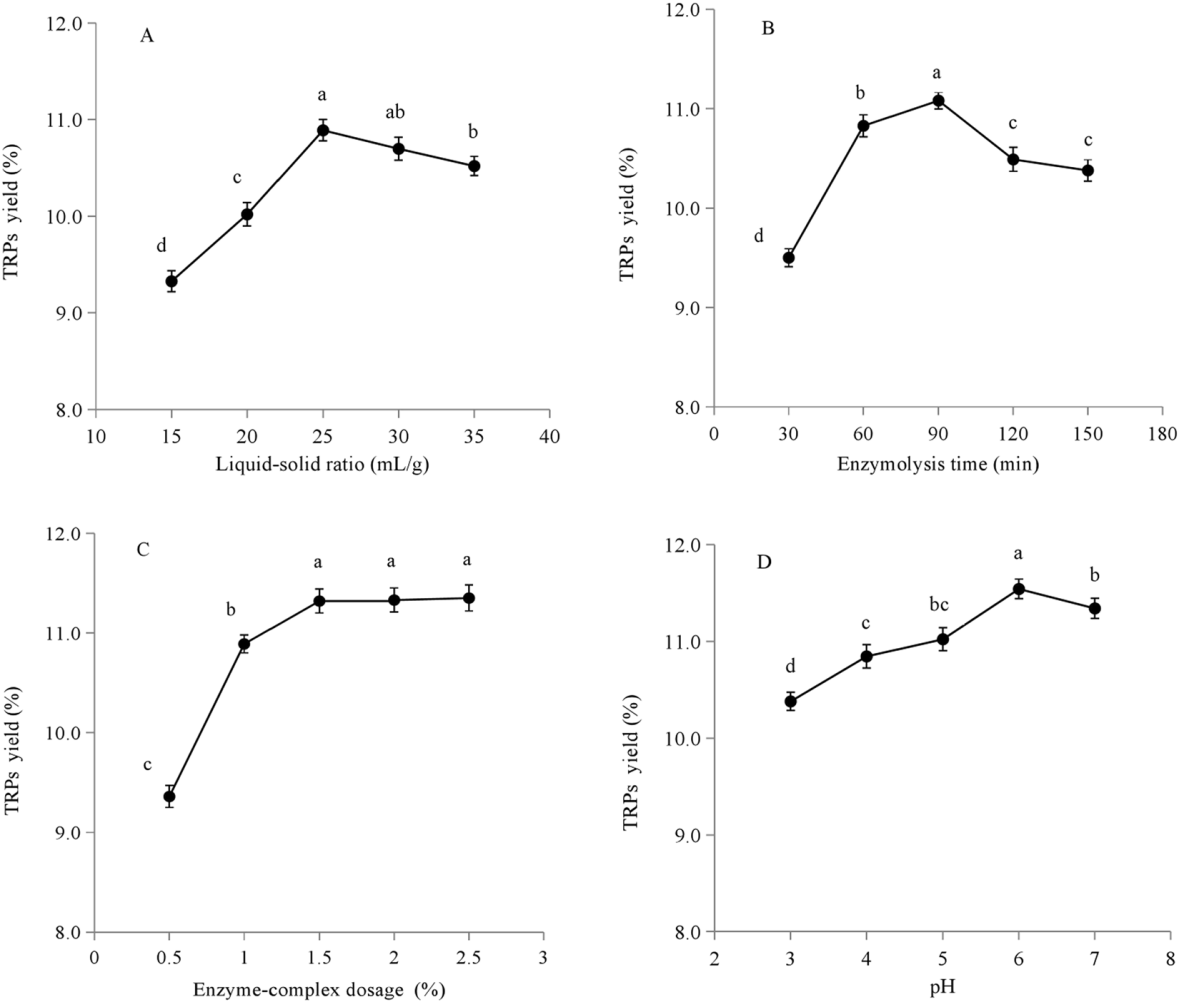
Effects of the four extraction parameters on the yield of TRPs: (**A**) liquid-solid ratio; (**B**) enzymolysis time; (**C**) enzyme-complex dosage; (**D**) pH. Different letters show significant difference (*P* < 0.05). Error bars presented the standard deviationas of means (n = 3). Duncan’s post hoc test was used in inmultiple means comparison procedure.

The results of single-factor-tests were shown in Fig. 4. Liquid-solid ratio, enzymolysis time, enzyme-complex dosage and pH all exhibited significant effects on the TRPs yield with the same trend. These results suggested that long enzymolysis time and high enzyme-complex dosage may lead to the decomposition of TRPs^33^. In addition, pH condition is a key factor ensuring optimal enzyme activity. Different pH conditions might lead to decreased or lost enzyme activities^34^. Moreover, excessive extraction solvent volume could increase the diffusion distance of TRPs from plant tissue, thereby inhibiting the dissolution of TRPs^35^. The maximum TRPs yields were obtained in certain ranges for the four single factors, including 20–30 mL/g for liquid-solid ratio, 60–120 min for enzymolysis time, 1–2% for enzyme-complex dosage and 5–7 for pH.

### Optimization of TRPs extraction by RSM

Based on the analysis of single-factor-tests, an RSM test was performed to obtain the optimal extraction parameters of TRPs. The TRPs yields were presented in Table 4. The predicted values were not significantly different from the corresponding actual values (*P* > 0.05). A multinomial regression equation was applied to demonstrate the relationship between the variables and the response. The multinomial regression equation was as follows:

$$\begin{array}{rcl}Y & = & -164.68+{\rm{4.67}}{X}_{1}+0.56{X}_{2}+22.29{X}_{3}+{\rm{21.96}}{X}_{4}\\  &  & -8.22\times {{\rm{10}}}^{-3}{X}_{1}{X}_{2}+{\rm{0.24}}{X}_{1}{X}_{3}-0.10{X}_{1}{X}_{4}\\  &  & -0.03{X}_{2}{X}_{3}+0.02{X}_{2}{X}_{4}-1.58{X}_{3}{X}_{4}\\  &  & -0.06{X}_{1}^{2}-2.13\times {{\rm{10}}}^{-3}{X}_{2}^{2}-5.50{X}_{3}^{2}-1.49{X}_{4}^{2}\end{array}$$

where *Y* is the TRPs yield, *X*_1_ is the liquid-solid ratio, *X*_2_ is the enzymolysis time, *X*_3_ is the enzyme-complex dosage, and *X*_4_ is the pH.

**Table 4:** Response and experimental design of enzyme-assisted extraction method to extract TRPs.

Test group	Liquid–solid ratio (*X*_1_) (mL/g)	Enzymolysis time (*X*_2_) (min)	Enzyme-complex dosage (*X*_3_) (%)	pH (*X*_4_)	TRPs yields (%)	
					Actual value	Predicted value
1	25	60	1.5	6	9.16	9.25
2	35	60	1.5	6	10.81	11.15
3	25	120	1.5	6	11.36	11.04
4	35	120	1.5	6	8.07	8.02
5	30	90	1	5	8.78	8.98
6	30	90	2	5	11.92	11.59
7	30	90	1	7	10.58	10.94
8	30	90	2	7	10.57	10.41
9	25	90	1.5	5	9.09	9.88
10	35	90	1.5	5	10.02	10.31
11	25	90	1.5	7	10.89	11.26
12	35	90	1.5	7	9.82	9.71
13	30	60	1	6	9.18	9.46
14	30	120	1	6	9.57	9.61
15	30	60	2	6	10.69	11.32
16	30	120	2	6	9.44	9.83
17	25	90	1	6	11.31	10.76
18	35	90	1	6	9.33	9.01
19	25	90	2	6	10.99	10.61
20	35	90	2	6	11.39	11.24
21	30	60	1.5	5	11.35	10.57
22	30	120	1.5	5	9.08	8.92
23	30	60	1.5	7	10.53	9.98
24	30	120	1.5	7	10.20	10.29
25	30	90	1.5	6	13.03	13.34
26	30	90	1.5	6	13.07	13.34
27	30	90	1.5	6	13.59	13.34
28	30	90	1.5	6	13.45	13.34
29	30	90	1.5	6	13.56	13.34

The predicted values were not significantly different from the corresponding actual values (*P* > 0.05).

The significance of the regression model was presented in Table 5. A low probability *P* value (<0.0001) suggested that the model was significant. The *P* value of the lack of fit was 0.0711, which was higher than 0.05, indicating that the model was valid^36^. The determination coefficient (*R*^2^) was 0.9413, suggesting a good correlation between the TRPs yield and the four independent variables. In addition, the *P*-value of linear coefficients (*X*_2_ and *X*_3_), the cross-product coefficients (*X*_1_*X*_2_, *X*_1_*X*_3_ and *X*_3_*X*_4_) and quadratic coefficients (*X*_1_^2^, *X*_2_^2^, *X*_3_^2^ and *X*_4_^2^) were less than 0.05, indicating the significant effects of the coefficients on the TRPs yield.

**Table 5:** ANOVA for the response surface quadratic model of TRPs yield.

Source	Sum of squares	df	Mean square	*F* value	*P* value	
Model	61.49	14	4.39	16.04	<0.0001*	significant
*X*_1_	0.94	1	0.94	3.43	0.0852	
*X*_2_	1.35	1	1.35	4.92	0.0436*	
*X*_3_	3.24	1	3.24	11.85	0.0040*	
*X*_4_	0.46	1	0.46	1.68	0.2164	
*X*_1_ *X*_2_	6.08	1	6.08	22.19	0.0003*	
*X*_1_ *X*_3_	1.42	1	1.42	5.19	0.0389*	
*X*_1_ *X*_4_	0.99	1	0.99	3.62	0.0779	
*X*_2_ *X*_3_	0.67	1	0.67	2.45	0.1399	
*X*_2_ *X*_4_	0.95	1	0.95	3.47	0.0835	
*X*_3_ *X*_4_	2.48	1	2.48	9.06	0.0094*	
*X*_1_^2^	15.85	1	15.85	57.86	<0.0001*	
*X*_2_^2^	23.77	1	23.77	86.80	<0.0001*	
*X*_3_^2^	12.26	1	12.26	44.78	<0.0001*	
*X*_4_^2^	14.40	1	14.40	52.58	<0.0001*	
Residual	3.83	14	0.27			
Lack of fit	3.54	10	0.35	4.84	0.0711	not significant
Pure error	0.29	4	0.073			
Cor total	65.33	28				
*R* ^2^	0.9413					
Adj *R*^2^	0.8826					
C.V%	4.88					

^*^P values < 0.05 indicate significant differences.

**Figure 5:**
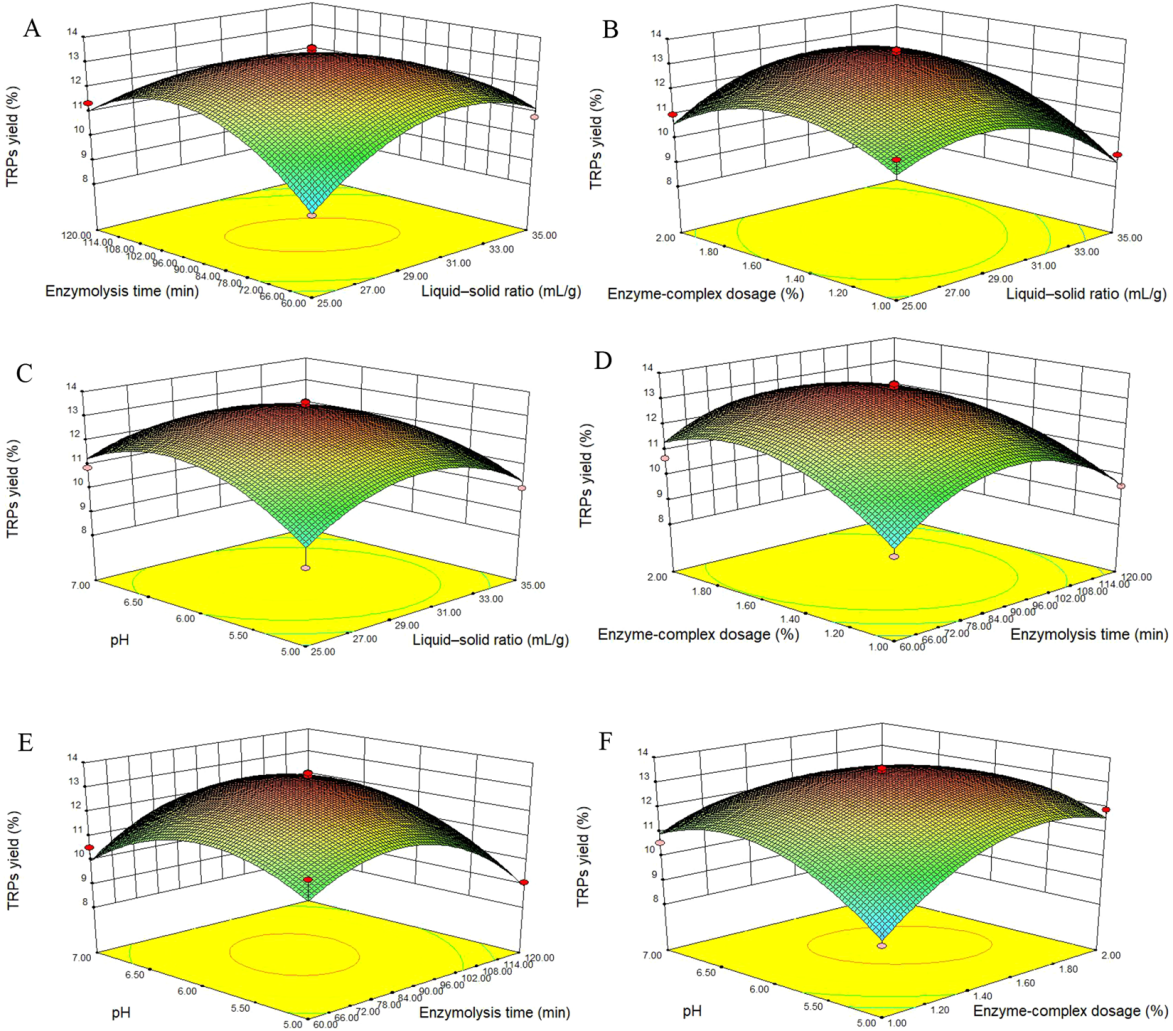
3D response surface plots showing the effects of liquid-solid ratio, enzymolysis time, enzyme-complex dosage, and pH on the extraction yield of TRPs and their mutual effects.

**Figure 6:**
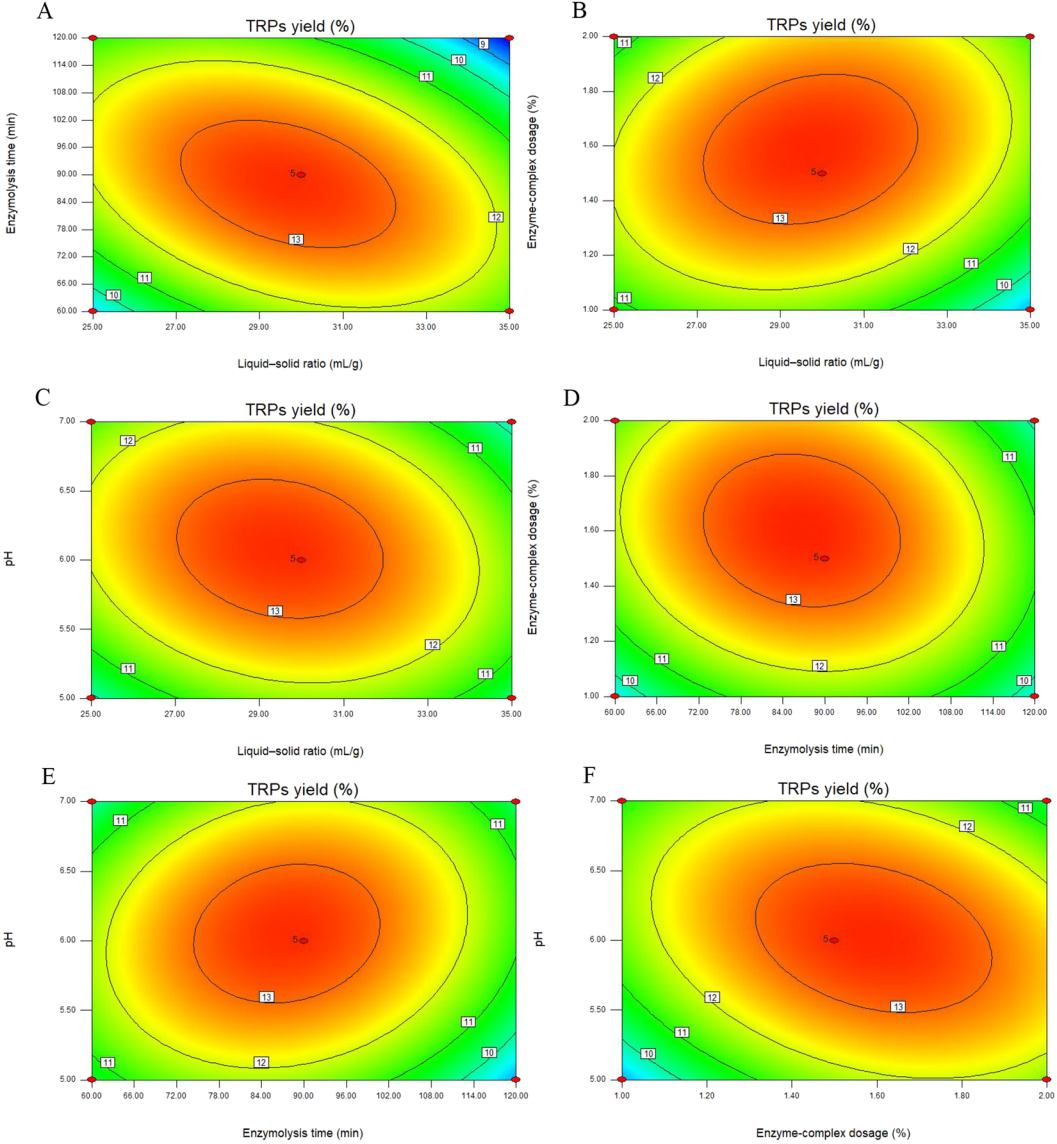
2D contour plots showing the effects of liquid-solid ratio, enzymolysis time, enzyme-complex dosage, and pH on the extraction yield of TRPs and their mutual effects.

The 3D response surface plots (Fig. 5) and 2D contour plots (Fig. 6) were the graphical presentations of the regression model. The visual interactions between the response data and the independent variables can be presented by the 3D response surface plots and 2D contour plots. The shapes of the 2D contour plots indicated the significance of the interactions between two variables. The circular contour plots suggest that the interactions between the two variables are not significant while the elliptical or saddle contour plots indicate that the interaction between the two variables are significant^18^. As shown in Fig. 6, the interactions of the variables (enzymolysis time and liquid-solid ratio, enzyme-complex dosage and liquid-solid ratio, and enzyme-complex dosage and pH) were significant (*P* < 0.05).

According to the regression model, the optimal extraction conditions of the EAE method to extract TRPs were liquid-solid ratio 29.96 mL/g, enzymolysis time 86.78 min, enzyme-complex dosage 1.6%, and pH 5.99. A predicted-maximum TRPs yield of 13.42% was obtained. To perform the extraction procedure more expediently, the actual extraction parameters were amended slightly as follows: liquid-solid ratio 30 mL/g, enzymolysis time 87 min, enzyme-complex dosage 1.6%, and pH 6. The verification experiments were repeated three times under the optimal extraction conditions, and the actual TRPs yield was 13.15 ± 0.12%, which was not significantly different from the predicted value of 13.42%. Therefore, the regression model obtained in this trial was effective in predicting TRPs yield. In recent years, the enzyme-assisted procedure is undoubtedly an emerging technology in polysaccharide extraction due to many advantages, including lower consumption of time, solvent and cost, higher properties of yields and purity, and good intervention on molecular structures^37^. A study was designed by Wang *et al*. to optimize the complex enzyme-assisted extraction parameters of the alfalfa polysaccharides using RSM design, and the optimal conditions were as follows: enzyme concentration of 2.5%, 2.0%, 3.0% (weight of alfalfa) of cellulase, papain and pectase, extraction temperature 52.7 °C, extraction pH 3.87, ratio of water to raw material 78.92 mL/g and extraction time 2.73 h. Under the optimal conditions, the experimental extraction yield of alfalfa polysaccharides was 5.05%^33^.

In conclusion, TRPs extracted by the EAE and UEAE methods had higher extraction yields among the four extraction methods (HWE, UAE, EAE and UEAE) used in this study, which might be observed because the enzyme complex (cellulase, papain and pectinase) could, separately or in conjunction with ultrasound, facilitate TRPs into the extraction solvents through enzymatic hydrolysis and cavitation effects. In addition, TRPs extracted by the EAE method had better antioxidant capacities, which might due to its higher content of uronic acid and lower molecular weight. Therefore, the enzyme-assisted extraction method was chosen to extract TRPs. According to the RSM analysis, the optimal extraction conditions of EAE to extract TPRs were liquid-solid ratio 30 mL/g, enzymolysis time 87 min, enzyme-complex dosage 1.6%, and pH 6, and a TRPs yield of 13.15% was obtained.

## Materials and Methods

Four different extraction methods (HWE, UAE, EAE and UEAE) were applied to extract TRPs. The physicochemical properties and antioxidant activities of TRPs were determined to select the suitable extraction method. In addition, RSM was performed to optimize the processing parameters of the suitable extraction method selected. All measurements were repeated in triplicate.

### Materials

*Trifolium repens* L. in bloom was obtained in Changchun City, China. The aerial portion of *Trifolium repens* L. was dried in a drier (101-2-BS, Shanghai Yuejin Medical Instrument co., LTD, Shanghai, China) at 50 °C for 10 h. Then, the dry plant material was smashed in a medicinal crusher (CS-700Y, Wuyi Haina Electric Appliance co., LTD, Wuyi, China) and sieved using a 40 mesh. Cellulase, papain, pectinase and vitamin C were purchased from Hefei Bomei Biotechnology Co., Ltd. (Hefei, China). DPPH was purchased from Tokyo Chemical Industry (Tokyo, Japan). DEAE-52 cellulose and ABTS were purchased from Blotopped (Beijing, China). Monosaccharide standards were purchased from Sigma Co., Ltd. (St. Louis, MO, USA). The remainder of the chemicals, including 1-phenyl-3-methyl-5-pyrazolone, glucose, phenol, Coomassie brilliant blue G250, bovine serum albumin, *m*-hydroxybiphenyl, sodium tetraborate, glucuronic acid, potassium peroxydisulfate, potassium ferricyanide, trichloroacetic acid, ferric trichloride and trifluoroacetic acid, were domestic and analytically pure.

### TRPs extraction with different methods

Four extraction techniques (HWE, UAE, EAE and UEAE) were performed following the procedures below, respectively, according to methods reported previously with some modifications^21,30^. Four corresponding polysaccharides, including HWE-TRPs, UAE-TRPs, EAE-TRPs and UEAE-TRPs, were obtained. Approximately 25 g of *Trifolium repens* L. powder was extracted with distilled water in a liquid material ratio of 20. HWE-TRPs was extracted in a water-bath at 90 °C for 90 min. UAE-TRPs was extracted in an ultrasonic device (KQ-100KDE, Kunshan Ultrasonic, China) working at a power of 100 W at 55 °C for 90 min. EAE-TRPs was extracted with enzyme complex (cellulase, papain and pectinase, each enzyme 1% (*w/w* raw material powder)) at 55 °C for 90 min. UEAE-TRPs was first extracted in an ultrasonic device working at a power of 100 W at 55 °C for 45 min, then extracted with the enzyme complex mentioned above at 55 °C for 45 min.

After extraction, the treatment processes, including the separation and concentration of the supernatant, the precipitation of polysaccharides, the removal of free proteins, the dialysis and drying of the polysaccharides solution, and the calculation of TRPs yield, were performed following the methods described previously^30^. The TRPs were first purified with a DEAE-52 cellulose column before further analysis (physicochemical characteristics and bioactivities)^11^.

### Physicochemical characteristics of TRPs extracted by different methods

The chemical compositions (the content of total polysaccharides, uronic acid, protein and moisture), pH and solubility of TRPs extracted by different methods were measured based on literature reports^30^. The molecular weight of TRPs was determined using gel filtration chromatography^11^. The monosaccharide composition of TRPs was analyzed using HPLC^38^.

### Antioxidant activities of TRPs extracted by different methods

The scavenging activities of DPPH radical and ABTS radical and the ferric reducing power were determined to evaluate the antioxidant activities of HWE-TRPs, UAE-TRPs, EAE-TRPs and UEAE-TRPs^35^. Vitamin C was the positive control.

### Extraction condition optimization of TRPs

After evaluating the experimental results of the physicochemical characteristics and activities of TRPs, EAE was selected as the better extraction method to extract TRPs. RSM was used to obtain the optimal extraction conditions of EAE to extract TRPs.

Approximately 25 g of *Trifolium repens* L. powder was extracted at 55 °C following the extraction procedure of ‘TRPs extraction with different methods’. A single-factor-test was performed to determine the preliminary range of variables, including *X*_1_ (liquid-solid ratio), *X*_2_ (enzymolysis time), *X*_3_ (enzyme-complex dosage) and *X*_4_ (pH). Then, the extraction conditions for the EAE method to extract TRPs were optimized by a BBD with three levels and four independent variables (*X*_1_, *X*_2_, *X*_3_ and *X*_4_) based on the results of single-factor experiments. The TRPs yield was treated as the response. The values of the experimental variables were shown in Table 4. Design Expert Software (8.0.6) was used to design the RSM experiment, perform the statistical analysis and fit the quadratic polynomial model. A quadratic polynomial model^39^ was performed to predict the optimal extraction conditions of EAE as follows:

$$Y={\beta }_{0}+\sum _{i=1}^{4}{\beta }_{i}{X}_{i}+\sum _{i=1}^{4}{\beta }_{ii}{X}_{i}^{2}+\sum \sum _{i < j=1}^{4}{\beta }_{ij}{X}_{i}{X}_{j}$$

where *Y* is the predicted response (TRPs yield); *X*_*i*_ and *X*_*j*_ are the variables; *β*_0_, *β*_*i*_, *β*_*ii*_ and *β*_*ij*_ are the regression coefficients for intercept, linear, quadratic, and interaction terms, respectively.

### Statistical analysis

The quantitative data results are presented as the mean ± standard deviation. One-way ANOVA of SPSS software (17.0) was used to perform the statistical comparisons followed by Duncan’s post hoc test. Significant differences were set at *P*-values < 0.05.

## Data Availability

All data generated or analyzed during this study are included in this published article.
